# Unraveling the interaction between stress and genetic risk in psychiatric disorders

**DOI:** 10.1192/j.eurpsy.2026.10408

**Published:** 2026-07-31

**Authors:** S. Penner-Goeke

**Affiliations:** Genes and Environment, Max Planck Institute of Psychiatry, Munich, Germany

## Abstract

**Abstract:**

Exposure to stress is a ubiquitous aspect of human life, but prolonged or traumatic stress is associated with an increased risk for a wide range of psychiatric disorders. However, not all individuals exposed to such stress develop psychiatric disorders, suggesting that additional factors must mediate risk. Indeed, a growing body of evidence suggests that stress exposure and genetic predisposition interact in complex gene–environment interactions. Much of what is known about these interactions in psychiatric disorders comes from epidemiological studies or from investigations focusing on interactions involving a single gene or a small set of genes. Methodological advances in recent years have made it possible to explore gene–environment interactions using novel approaches that provide insight into their polygenic nature as well as into potential mechanisms through which these interactions act, including epigenetic processes. This session will examine the current state of the field, identify existing limitations, and discuss technological advancements that facilitate focused mechanistic investigations into the interplay between stress and genetic risk.

Specifically, an example will be presented illustrating how novel molecular genetic tools can be used to identify gene–environment interactions and map them onto stress physiology and stress-related neuroimaging phenotypes in a human cohort. This case will serve as an example of how multimodal data—ranging from molecular genetic readouts to human phenotypic measures—can be integrated to better understand stress physiology across multiple levels.

**Disclosure of Interest:**

None Declared

